# Mitochondrial Network Genes in the Skeletal Muscle of Amyotrophic Lateral Sclerosis Patients

**DOI:** 10.1371/journal.pone.0057739

**Published:** 2013-02-28

**Authors:** Camilla Bernardini, Federica Censi, Wanda Lattanzi, Marta Barba, Giovanni Calcagnini, Alessandro Giuliani, Giorgio Tasca, Mario Sabatelli, Enzo Ricci, Fabrizio Michetti

**Affiliations:** 1 Institute of Anatomy and Cell Biology, School of Medicine, Università Cattolica del Sacro Cuore, Rome, Italy; 2 Department of Technologies and Health, Istituto Superiore di Sanità, Rome, Italy; 3 Department of Environment and Health, Istituto Superiore di Sanità, Rome, Italy; 4 Don Carlo Gnocchi Onlus Foundation, Milan, Italy; 5 Institute of Neurology, Università Cattolica del Sacro Cuore, School of Medicine, Rome, Italy; University of Rome La Sapienza, Italy

## Abstract

Recent evidence suggested that muscle degeneration might lead and/or contribute to neurodegeneration, thus it possibly play a key role in the etiopathogenesis and progression of amyotrophic lateral sclerosis (ALS). To test this hypothesis, this study attempted to categorize functionally relevant genes within the genome-wide expression profile of human ALS skeletal muscle, using microarray technology and gene regulatory network analysis. The correlation network structures significantly change between patients and controls, indicating an increased inter-gene connection in patients compared to controls. The gene network observed in the ALS group seems to reflect the perturbation of muscle homeostasis and metabolic balance occurring in affected individuals. In particular, the network observed in the ALS muscles includes genes (PRKR1A, FOXO1, TRIM32, ACTN3, among others), whose functions connect the sarcomere integrity to mitochondrial oxidative metabolism. Overall, the analytical approach used in this study offer the possibility to observe higher levels of correlation (i.e. common expression trends) among genes, whose function seems to be aberrantly activated during the progression of muscle atrophy.

## Introduction

Amyotrophic lateral sclerosis (ALS) is classically defined as a motor neuron disorder, characterized by the progressive degeneration of upper and lower motor neurons, leading to muscle atrophy and corresponding loss of muscle strength. ALS is eventually lethal within 3-to-5 years after the symptom onset, due to the involvement of respiratory muscles. Although muscle atrophy has been originally considered a secondary direct consequence of neurodegeneration, new pathogenetic hypotheses, during the last decade, have been suggesting a primary role for the events occurring at the post-synaptic site, i.e. in the skeletal muscle. Damage of the neuromuscular junction (NMJ), along with skeletal muscle degeneration and atrophy, indeed precede neuronal degeneration in the ALS SOD1 mouse model [1], [2]. This supports the notion that muscle degeneration may lead and/or contribute to neurodegeneration and play a key role in the cause and/or progression of ALS [3], [4].

Skeletal muscle of ALS patients and ALS mice presents severe atrophy [5] and has considerable mitochondrial disruption and dysfunction, indicated by the deficiency of key respiratory chain enzymes [6]–[8]. It was recently demonstrated that the disruption of the mitochondrial network genes enhances skeletal muscle atrophy programs [9], [10]. This evidence indicates that a perturbation in the homeostasis of the molecular network involved in the maintenance of skeletal muscle mitochondrial biogenesis should occur during the pathogenesis and progression of ALS [10].

Though, the hypothesis of the skeletal muscle as the primary damaged site in ALS pathogenesis is still pending, due to the dearth of evidences obtained in human tissues, which represent the unique clinically-relevant model to study sporadic ALS.

As a tool to investigate the molecular scenario occurring at the post-synaptic site in ALS injured muscles, we used microarray-based genome-wide expression profiling. In order to obtain original hints toward the functional interpretation of the wide resulting dataset we used a system biology approach, based on gene regulatory networks, to analyze the changes in between gene expression correlation structure occurring in the affected tissue [11].

Microarray data analysis has been classically based on supervised statistical methods, enabling to compare gene-by-gene differential expression. This approach tends to consider genes as independent functional units, regardless of the multifactorial regulation of gene expression acting in biological systems [12]–[14]. In addition, given the high dimensionality of the microarray experiments compared to the number of tested samples/individuals, it can be severely biased by chance correlation effect [15]. On this regard, gene networks describe the connections existing between genes that are involved in the same biological process and are used to identify functional modules (i.e. subsets of genes that regulate each other with multiple interactions, but have few interactions with other genes outside the subset).

Therefore, the analytical strategy proposed in this study, allows identifying the expression signature of the atrophic skeletal muscle, based both on differential gene expression and on gene correlation networks.

## Materials and Methods

### Samples Collection

#### Ethics statement

The Ethics Committee of the Università Cattolica del Sacro Cuore, School of Medicine (Rome, Italy) approved this study. Written informed consent was obtained from all patients.

#### Patients and specimens

A case-control study was performed comparing 7 ALS patients with 7 age- and sex-matched healthy controls (table 1). The ALS sample was represented by 4 males and 3 females, aged 55-to-73; mean and median age was 64 in both the patients and the control group. ALS diagnosis was performed according to the revised El Escorial criteria. The control sample was selected among patients undergoing orthopedic surgery for traumatic injury and without positive history for muscle weakness, nor any neurological disorder. All patients were of Caucasian origin. Detailed clinical information of the individuals enrolled in the study is provided in table 1. A skeletal muscle specimen was collected through an open biopsy performed either in the deltoid muscle (4 ALS patients and all controls) or in the quadriceps (3 ALS patients).

**Table 1:** Differentially expressed genes list.

Case ID	Group	Site of onset	Gender	Age (years)^1^	Duration^2^	ALSFRS-R	Biopsied muscle
**1**	**control**	**−**	Male	59	**−**	**−**	deltoid
**2**	**control**	**−**	Female	62	**−**	**−**	deltoid
**3**	**control**	**−**	Male	66	**−**	**−**	deltoid
**4**	**control**	**−**	Female	60	**−**	**−**	deltoid
**5**	**control**	**−**	Female	64	**−**	**−**	deltoid
**6**	**control**	**−**	Female	70	**−**	**−**	deltoid
**7**	**control**	**−**	Male	65	**−**	**−**	deltoid
**8**	**ALS**	Spinal	Male	73	na	34	quadriceps
**9**	**ALS**	Spinal	Female	72	11	**33**	quadriceps
**10**	**ALS**	Spinal	Female	59	13	**35**	deltoid
**11**	**ALS**	Bulbar	Male	54	8	**38**	deltoid
**12**	**ALS**	Spinal	Male	72	18	**35**	deltoid
**13**	**ALS**	Spinal	Female	55	9	**32**	deltoid
**14**	**ALS**	Bulbar	Male	64	na	**36**	quadriceps

1 Ages of patients and controls are not significantly different (p: 0.2648).

2 Time from symptom onset to muscle biopsy.

The tissue specimens were stored in liquid nitrogen upon collection and subsequently used for RNA isolation.

#### RNA isolation and microarray analysis

Total RNA was isolated from frozen tissue specimens using pestel homogenization, TRIzol protocol (Invitrogen, Carlsbad, CA, USA), and further on-column purification as previously described [16]. The yield, quality and integrity of RNA were determined using the Agilent 2100 Bioanalyzer (Agilent Technologies, Palo Alto, CA, USA) as previously described [17]. The resulting total RNA was then used to create the biotin-labeled library to be hybridized on GeneChip Human Genome Focus Array (Affymetrix, Santa Clara, CA, USA), as already described elsewhere [18].

### Microarray Data Analysis

**Figure 1:**
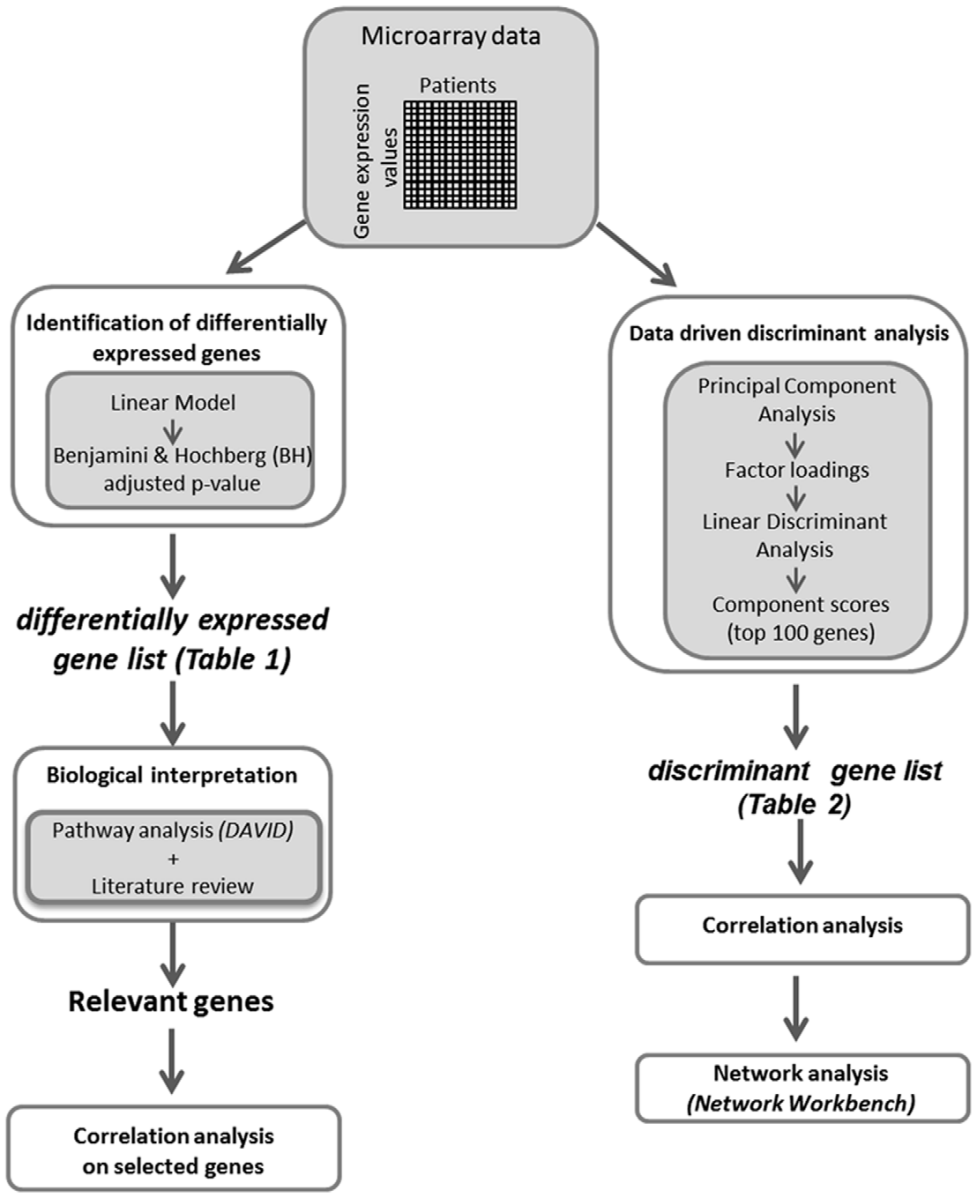
Experimental design. The flowchart schematizes the experimental steps of the statistical analysis of microarray data.

The overall data analysis process involved to distinct level of analysis, according to the flowchart depicted in figure 1.

#### Gene expression data analysis – differentially expressed gene list

The CEL files resulting from the hybridization were analyzed using the oneChannelGUI 1.6.5 bioinformatics tool [19]. Gene-level calculation was performed by Robust Multichip Average [20] and normalization by quantile sketch [21], [22]. A data table (rma), together with the relative cel files and relevant information about the experiment, is available at http://www.ncbi.nlm.nih.gov/geo accession #GSE 41414.

Quality control of samples was assessed by unsupervised multidimensional scaling (MDS) analysis on all probeset intensity values, in order to assess the segregation efficiency of samples. In the MDS analysis samples are positioned in a tridimensional space on the basis of first three principal components of variability [17].

To assess differential gene expression levels, an empirical Bayes method was employed [23]. The intensity values were filtered using an inter quantile range (IQR) = 0,25, to remove invariant probe-set on the basis of their distribution in the array under analysis.

A hierarchical linear modeling approach was then used to identify differentially expressed genes. This method is based on fitting a linear model to estimate the variability of the studied data. A Benjamini & Hochberg (BH)-adjusted p-value was calculated and a p≤0.05 cut-off was set. The resulting gene list was then annotated according to the Gene Ontology (GO) database (www.geneontology.org). This allowed assigning a category to each gene in the list, according to three defined “ontologies” (i.e. terms representing gene product properties): cellular component, biological process and molecular function.

The expression of selected genes was quantified in real time PCR to obtain an independent validation of microarray data. Real time PCR was carried out as previously described elsewhere [24].

#### Principal component analysis – discriminant gene list

The discrimination has been performed using an *a posteriori* unsupervised approach relying on the application of principal component analysis (PCA) of the gene expression profiles, where the genes represent the statistical units and the patients are used as variables. This inversion leads to a better statistically conditioned approach, since genes largely outnumber patients [25]. Moreover this approach minimizes the variability in gene expression profiles due to tissue type.

The PCA has been used to extract a list of genes that best discriminate the two groups (patients and controls), adopting a correlation-based approach, devoid of any overfitting/chance significance risk. Indeed PCA is an unsupervised method, which analyses the differences in gene expression profiles between patients and controls, related to the very small part of the data variability [13], [25], [26]. To this aim, the principal components were extracted from a matrix having genes as statistical units and patients as variables. Raw data from the entire gene set were used without any a priori selection. PCA projects the initial space spanned by the different samples into a new derived space whose axes (principal components, PCs) are each other orthogonal. This allows for a direct, unbiased normalization of the data field, where the ‘shared variance’ is accounted for by the first principal component. The minor components (from second component onward) keep trace of the relevant differences among samples.

The analysis of such a small difference was performed in terms of factor loadings (FL, correlation coefficients between original variables and components) and scores.

The space of component loadings represents different individuals in terms of similarities in the gene expression space. Fls are the correlation coefficients between original variables and components; given in our analysis the variables correspond to the individuals, FLs allow for a quantification of the weight of each patient and healthy sample on each principal component.

The FLs were then analyzed by a linear discriminant analysis to find out whether and which FL allows for a separation of the data set into patients and controls.

The component scores represent the contribution of the observations (statistical units, that are the gene expression values in this case) to the principal component. Thus for each gene expression value we calculated a score, with the component having a relevant discriminant power. This procedure allowed for a biological association of components to groups of genes and thus permitted a biological interpretation of the obtained discrimination.

In the space of component scores each gene is defined, and it is possible to order genes on the basis of their scores with components endowed with a relevant discriminant power.

#### Correlation analysis

The correlation existing between two genes in the population, based on their expression trends, was calculated using the Pearson correlation coefficient.

The quantification of the correlation between genes can be used to analyze the link between any pair of genes. In this work we use this information both to construct the gene network and to analyze the link between selected genes.

#### Network analysis

The 100 genes that best discriminate patients from controls, sorted according to the principal component scores, were then analyzed in terms of gene correlation network.

To this aim, the intergenic correlation structure was represented as a network, where the nodes were genes connected by edges. The genes were connected if the correlation coefficient between their expression values in the population was higher than a given threshold (i.e. the trend of expression values over the population is similar) [27].

The choice of the threshold is not trivial. Many biologically relevant connections could not be included in such network if the threshold is too high, while lowering the correlation threshold will significantly increase the number of potential links, including many random ones. The choice of the threshold has been made according to the results obtained from surrogate data analysis, which allows quantifying the connections detected by chance or due to noise [27]. Surrogate data effectively destroy any correlation between pair of gene expression values across the population, by randomly shuffling the gene expression values, i.e., by reassigning each gene expression value to a different individual. On the other hand, if there are no correlation, randomly shuffling the expression values across individuals will not alter the correlation value: any associations should still be small and attributable to chance. To this aim, individuals in the population are indexed from 1 to n. The data are shuffled by computing a random permutation of the indices 1,..., n and assigning the i^th^ gene expression value to the individual whose index is given by the i^th^ element of the permutation. The shuffled data were then analyzed in terms of number of connections, given a certain value of correlation threshold. To increase the robustness of the method, 1000 realizations of surrogate data have been generated. The threshold was set by computing the average number of resulting connections for the surrogate data. Particularly, given N genes, the number of potential connections is N*(N−1)/2 (for 100 genes it results in 4950 potential connections). Based on these considerations, the threshold for the correlation coefficient was set so that the number of connection for surrogate data was at least 10% lower than that obtained for original data, in this case 0.95. The wiring pattern of the networks was set on the basis of their actual correlation coefficient, as computed over the entire population.

The global topological structure of the networks was analyzed by using the Network Workbench, a large-scale network analysis, modeling and visualization toolkit for biomedical, social science, and physics research (freely downloadable at http://nwb.cns.iu.edu).

Besides the visual inspection of the networks, the functional connection of the genes was quantified by computing the average degree (AD) of the gene network. The AD denotes the number of links that connect a node to the rest of the network and is calculated as AD = 2*C/N, where C is the number of connections and N is the number of genes [28].

#### Functional categorization

Both the gene expression list and the network-based gene list have been functionally categorized using the Database for Annotation, Visualization, and Integrated Discovery (DAVID; http://david.abcc.ncifcrf.gov/) [29]. The algorithms implemented in this software allow identifying over-represented gene ontology (GO) terms with respect to the total number of genes assayed and annotated. To this aim, DAVID applies a modified Fisher exact test, to establish if the proportion of genes falling into an annotation category significantly differs from the background group of genes. In addition, this tool enables the fine mapping of genes within well-defined signaling and/or metabolic pathways, classified in the Kyoto Encyclopedia of Genes and Genomes (KEGG) database (www.genome.jp/kegg/). The KEGG mapping tool was employed for the functional categorization of the gene regulatory networks.

For this purpose, AffyGene IDs, corresponding to the genes in the selected list, were used as queries and the whole set of genes represented on the array was used as the background group. A false discovery rate (FDR) ≤0.05 was set.

## Results

### Differentially Expressed Gene List

**Figure 2:**
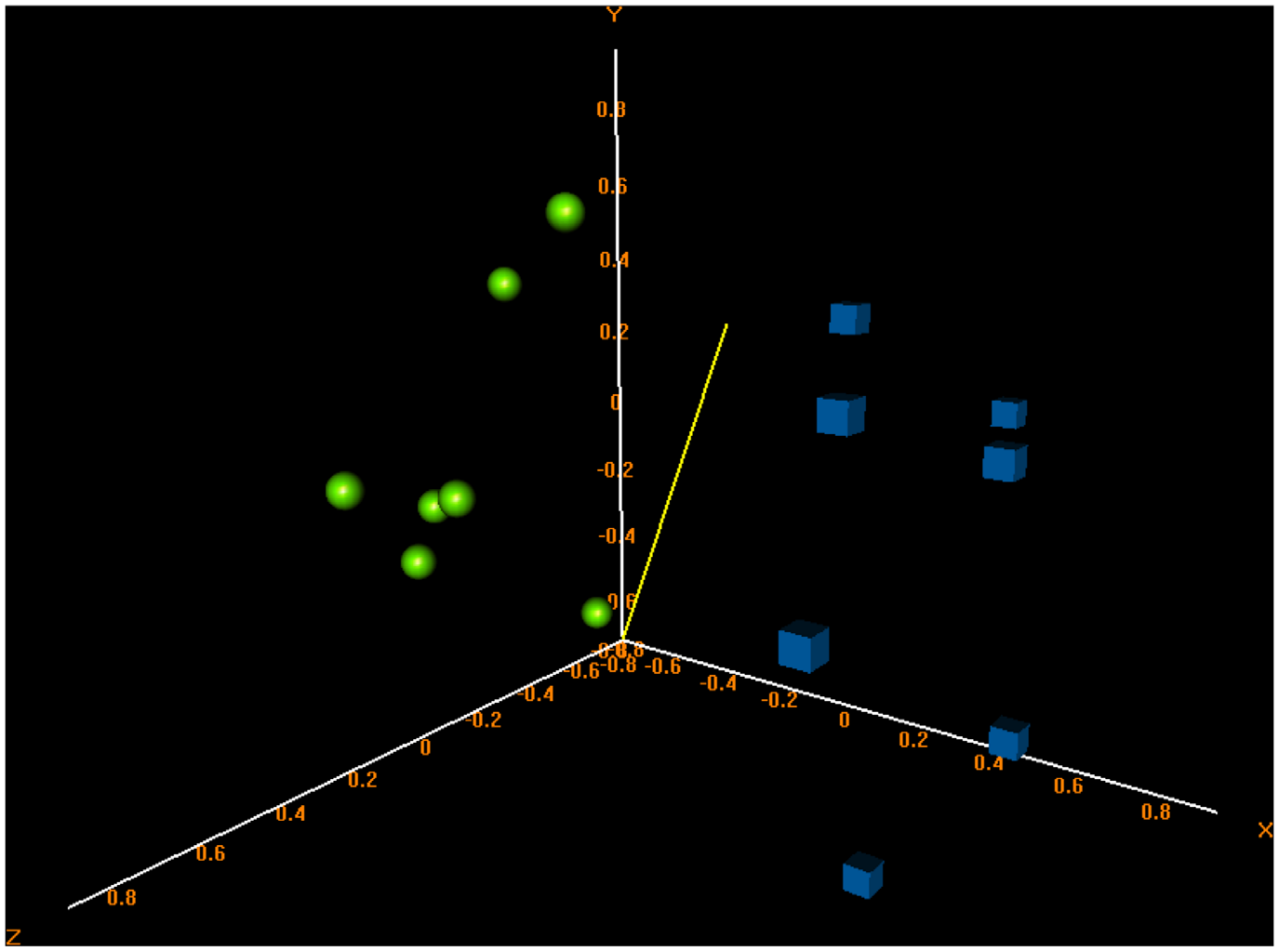
Multidimensional scaling of expression data. MDS of the expression profiles of ALS and controls shows the correct segregation of samples (see text for details). ALS samples are represented by spheres and controls by cubes.

The MDS analysis based on the expression level of all the 8793 probesets spotted on the array was performed in order to evaluate the segregation of the sample groups. As illustrated in figure 2, the analysis showed the efficient segregation of ALS samples from control samples.

**Figure 3:**
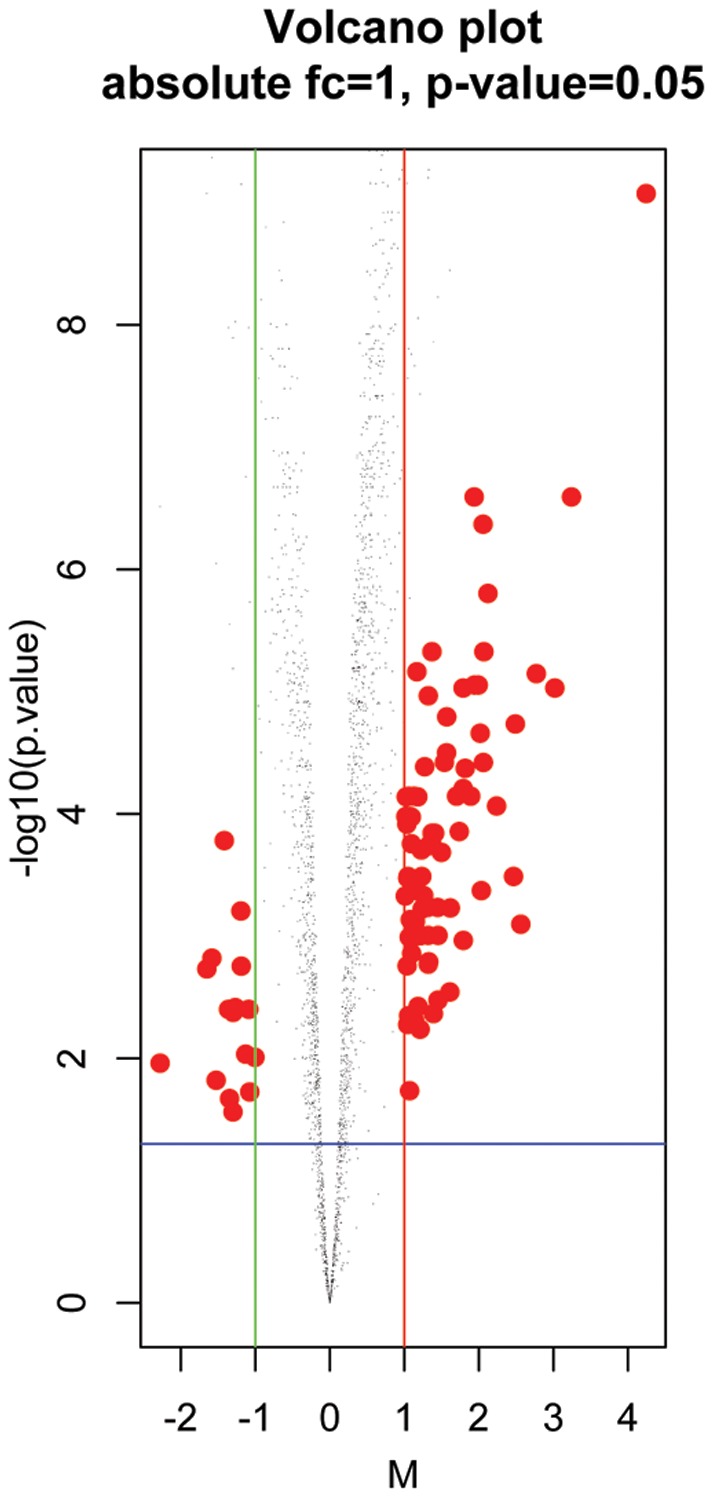
Volcano plot. This representation of data resulting from microarray analysis compares the size of the fold change with the statistical significance level. x-axis: log_2_ of FC (fold change of differential gene expression between the ALS patients and controls); y-axis: corresponding p-value resulting from t-test used to measure the significance of the difference between samples in groups.

To identify differentially expressed genes, the dataset was filtered by IQR, which allowed obtaining 2478 transcript clusters out of 8793. Thereafter, an absolute log_2_-fold change ≥1 and a BH-corrected P-value ≤0.05 were used as cutoffs (figure 3). This allowed identifying 96 differentially expressed transcripts (table 1), including 16 downregulated and 80 upregulated genes in the ALS patient samples compared to controls.

According to the GO annotations, the gene list included functional categories related to the skeletal muscle structure and metabolism (table 2). In particular, three downregulated genes (namely PGAM2, FBP2 and ENO3, all muscle-specific) and an upregulated one (PFKP), encode enzymes involved in glycolysis and gluconeogenesis. Also, nine transcripts included in the gene list are active during skeletal muscle contraction and skeletal system development. These latter included: four distinct myosin chains (MYH3, MYH8, MYL3, MYL5), myogenin (MYOG), the myogenic factor 6 (MYF6) and collagens (COL1A1, COL1A2, COL3A1). In addition, the forkhead box O1 (FOXO1) gene, belonging to the FoxO family members present in skeletal muscle, and the cAMP-dependent protein kinase regulatory subunit RI1 alpha (PRKAR1A) were significantly up-regulated (table 2).

**Table 2:** Differentially expressed genes list.

Probe Set ID	Gene Title	Gene Symbol	P.Value	FC^a^
206633_at	cholinergic receptor, nicotinic, alpha 1 (muscle)	CHRNA1	<0,0001	18,96
203725_at	growth arrest and DNA-damage-inducible, alpha	GADD45A	<0,0001	9,47
208623_s_at	ezrin	EZR	<0,0001	3,83
201012_at	annexin A1	ANXA1	<0,0001	4,16
206559_x_at	eukaryotic translation elongation factor 1 alpha 1	EEF1A1	<0,0001	4,19
201037_at	phosphofructokinase, platelet	PFKP	<0,0001	2,59
202237_at	nicotinamide N-methyltransferase	NNMT	<0,0001	8,11
37996_s_at	dystrophia myotonica-protein kinase	DMPK	<0,0001	3,46
204892_x_at	eukaryotic translation elongation factor 1 alpha 1	EEF1A1	<0,0001	4,35
217755_at	hematological and neurological expressed 1	HN1	<0,0001	2,25
209288_s_at	CDC42 effector protein (Rho GTPase binding) 3	CDC42EP3	<0,0001	6,83
204802_at	Ras-related associated with diabetes	RRAD	<0,0001	3,71
204225_at	histone deacetylase 4	HDAC4	<0,0001	3,25
207024_at	cholinergic receptor, nicotinic, delta	CHRND	<0,0001	2,19
211340_s_at	melanoma cell adhesion molecule	MCAM	<0,0001	2,09
201289_at	cysteine-rich, angiogenic inducer, 61	CYR61	<0,0001	3,98
203571_s_at	chromosome 10 open reading frame 116	C10orf116	<0,0001	3,84
209014_at	melanoma antigen family D, 1	MAGED1	<0,0001	2,50
202769_at	cyclin G2	CCNG2	<0,0001	2,97
202284_s_at	cyclin-dependent kinase inhibitor 1A (p21, Cip1)	CDKN1A	<0,0001	5,62
202310_s_at	collagen, type I, alpha 1	COL1A1	<0,0001	4,05
218718_at	platelet derived growth factor C	PDGFC	<0,0001	2,96
202403_s_at	collagen, type I, alpha 2	COL1A2	<0,0001	2,90
205145_s_at	myosin, light chain 5, regulatory	MYL5	<0,0001	4,17
208682_s_at	melanoma antigen family D, 2	MAGED2	<0,0001	2,42
220359_s_at	cAMP-regulated phosphoprotein, 21 kDa	ARPP21	<0,0001	3,52
222162_s_at	ADAM metallopeptidase with thrombospondin type 1 motif, 1	ADAMTS1	<0,0001	3,46
207282_s_at	myogenin (myogenic factor 4)	MYOG	<0,0001	2,27
200782_at	annexin A5	ANXA5	<0,0001	2,04
205132_at	actin, alpha, cardiac muscle 1	ACTC1	<0,0001	4,72
201666_at	TIMP metallopeptidase inhibitor 1	TIMP1	0,00011	2,11
203186_s_at	S100 calcium binding protein A4	S100A4	0,00011	2,05
204257_at	fatty acid desaturase 3	FADS3	0,00011	2,03
209785_s_at	phospholipase A2, group IVC	PLA2G4C	0,00011	2,13
202157_s_at	CUGBP, Elav-like family member 2	CELF2	0,00012	2,05
203579_s_at	solute carrier family 7, member 6	SLC7A6	0,00014	3,33
206703_at	cholinergic receptor, nicotinic, beta 1 (muscle)	CHRNB1	0,00014	2,65
200665_s_at	secreted protein, acidic, cysteine-rich (osteonectin)	SPARC	0,00014	2,59
201368_at	zinc finger protein 36, C3H type-like 2	ZFP36L2	0,00017	2,58
202724_s_at	forkhead box O1	FOXO1	0,00018	2,14
217728_at	S100 calcium binding protein A6	S100A6	0,00018	2,50
202854_at	hypoxanthine phosphoribosyltransferase 1	HPRT1	0,00020	2,34
201300_s_at	prion protein	PRNP	0,00021	2,82
34471_at	myosin, heavy chain 8, skeletal muscle, perinatal	MYH8	0,00033	5,52
203232_s_at	ataxin 1	ATXN1	0,00033	2,35
202133_at	WW domain containing transcription regulator 1	WWTR1	0,00033	2,07
203156_at	A kinase (PRKA) anchor protein 11	AKAP11	0,00033	2,06
201141_at	glycoprotein (transmembrane) nmb	GPNMB	0,00034	2,16
208683_at	calpain 2, (m/II) large subunit	CAPN2	0,00041	2,16
205940_at	myosin, heavy chain 3, skeletal muscle, embryonic	MYH3	0,00042	4,09
212271_at	mitogen-activated protein kinase 1	MAPK1	0,00042	2,14
204039_at	CCAAT/enhancer binding protein (C/EBP), alpha	CEBPA	0,00047	2,40
200603_at	protein kinase, cAMP-dependent, regulatory, type I, alpha	PRKAR1A	0,00047	2,02
219825_at	cytochrome P450, family 26, subfamily B, polypeptide 1	CYP26B1	0,00058	2,73
211962_s_at	zinc finger protein 36, C3H type-like 1	ZFP36L1	0,00058	2,49
211161_s_at	collagen, type III, alpha 1	COL3A1	0,00059	3,07
201417_at	SRY (sex determining region Y)-box 4	SOX4	0,00060	2,36
209118_s_at	tubulin, alpha 1a	TUBA1A	0,00073	2,11
201530_x_at	eukaryotic translation initiation factor 4A1	EIF4A1	0,00076	2,22
206717_at	myosin, heavy chain 8, skeletal muscle, perinatal	MYH8	0,00080	5,91
201761_at	methylenetetrahydofolate dehydrogenase 2	MTHFD2	0,00099	2,74
202995_s_at	fibulin 1	FBLN1	0,00099	2,49
201744_s_at	lumican	LUM	0,00100	2,31
202598_at	S100 calcium binding protein A13	S100A13	0,00102	2,18
200059_s_at	ras homolog gene family, member A	RHOA	0,00102	2,09
206306_at	ryanodine receptor 3	RYR3	0,00108	3,46
206059_at	zinc finger protein 91	ZNF91	0,00139	2,14
211985_s_at	calmodulin 1 (phosphorylase kinase, delta)	CALM1	0,00162	2,51
212670_at	elastin	ELN	0,00170	2,50
200696_s_at	gelsolin	GSN	0,00175	2,05
212099_at	ras homolog gene family, member B	RHOB	0,00287	3,05
201329_s_at	v-ets erythroblastosis virus E26 oncogene homolog 2	ETS2	0,00334	2,74
200866_s_at	prosaposin	PSAP	0,00376	2,27
201426_s_at	vimentin	VIM	0,00430	2,62
204719_at	ATP-binding cassette, sub-family A (ABC1), member 8	ABCA8	0,00447	2,08
206372_at	myogenic factor 6 (herculin)	MYF6	0,00461	2,13
202838_at	fucosidase, alpha-L- 1, tissue	FUCA1	0,00493	2,20
207992_s_at	adenosine monophosphate deaminase 3	AMPD3	0,00529	2,06
201946_s_at	chaperonin containing TCP1, subunit 2 (beta)	CCT2	0,00578	2,32
208607_s_at	serum amyloid A1/serum amyloid A2	SAA1/SAA2	0,01840	2,10
205589_at	myosin, light chain 3, alkali; ventricular, skeletal, slow	MYL3	0,00017	**−**2,66
203766_s_at	leiomodin 1 (smooth muscle)	LMOD1	0,00062	**−**2,28
205738_s_at	fatty acid binding protein 3, muscle and heart	FABP3	0,00151	**−**2,99
202428_x_at	diazepam binding inhibitor	DBI	0,00176	**−**2,27
205736_at	phosphoglycerate mutase 2 (muscle)	PGAM2	0,00185	**−**3,14
218818_at	four and a half LIM domains 3	FHL3	0,00384	**−**2,39
205330_at	meningioma (disrupted in balanced translocation) 1	MN1	0,00397	**−**2,55
219983_at	HRAS-like suppressor	HRASLS	0,00397	**−**2,11
205478_at	protein phosphatase 1, regulatory (inhibitor) subunit 1A	PPP1R1A	0,00421	**−**2,45
204483_at	enolase 3 (beta, muscle)	ENO3	0,00924	**−**2,17
216733_s_at	glycine amidinotransferase	GATM	0,00981	**−**2,01
206891_at	actinin, alpha 3	ACTN3	0,01094	**−**4,82
206844_at	fructose-1,6-bisphosphatase 2	FBP2	0,01511	**−**2,97
203824_at	tetraspanin 8	TSPAN8	0,01882	**−**2,10
204783_at	myeloid leukemia factor 1	MLF1	0,02132	**−**2,53
208691_at	transferrin receptor (p90, CD71)	TFRC	0,02744	**−**2,28

a FC: Fold Change.

To further investigate the possible transcriptional interplay for FOXO1 and PRKAR1A the correlation existing between each of the two genes and all the other genes in the dataset was analyzed. The correlation coefficient between FOXO1 and PRKAR1A resulted statistically significant (0.89), suggesting that the expression trends of these two genes were closely correlated in the ALS group. In addition, the gene showing the highest degree of correlation with PRKAR1A in the ALS group was the Tripartite motif containing 32 (TRIM32) (correlation coefficient = 0.97). The same gene showed a lower but still significant correlation with FOXO1 (correlation coefficient = 0.85).

The results of quantitative real time PCR, performed to amplify selected genes from the gene list, confirmed the trends of gene expression obtained with microarray analysis (figure S1).

### Biological Interpretation of the Differentially Expressed Gene List

The functional analysis of the gene list was accomplished using DAVID annotation tool, which allowed identifying the most significant biological functions in the data set (FDR ≤0.05). In particular, the biological processes were linked to: skeletal muscle development/contraction and response to organic substance, consistently with previous data [30]. The most significant cellular component and molecular function categories involved in this analysis were correlated to “actin cytoskeleton” and “cytoskeletal protein binding”, respectively. The most represented ‘biological pathway’ categories were represented by focal adhesion, regulation of actin cytoskeleton and glycolysis/gluconeogenesis. Complete results are reported in table S1.

### Discriminant Gene List

The PCA performed on the microarray dataset allowed categorizing the principal components (PCs) of data variability. Usually, the first PC (PC1) accounts for more than 97% of the total variability and represents the ‘tissue type attractor’ [14], i.e. the typical profile of the analyzed tissue. The vast part of variance explained by PC1 in this study confirmed the samples homogeneity, as a result of the careful sample selection carried out based on patients’ clinical features (Table 1).

The FL that further best discriminated between ALS patients and controls, corresponded to the second PC. Hence, the scores related to the PC2 were used to generate the list of most discriminant genes. The 100 genes with the highest FL scores (in absolute value) for the PC2 are showed in table 3, sorted in descending order. Common genes featured in both the differentially expressed gene list (table 2) and the discriminant gene list (table 3) were 59 and are reported in bold in table 3.

**Table 3:** Discriminant genes list.

Order	Gene	Gene ontology	MT^a^	SM^b^	Order	Gene	Gene ontology	MT^a^ T	MU
**1**	**ACTN3**	actinin, alpha 3	X	X	**51**	**MAGED1**	melanoma antigen family D, 1		X
**2**	**PGAM2**	phosphoglycerate mutase 2		X	**52**	**HPRT1**	hypoxanthine phosphoribosyltransferase 1	X	
**3**	**MYL3**	myosin, light chain 3, alkali; ventricular, skeletal, slow		X	**53**	–	–		
**4**	**MLF1**	myeloid leukemia factor 1			**54**	**ABCA8**	ATP-binding cassette, sub-family A (ABC1), member 8		
**5**	**FBP2**	fructose-1,6-bisphosphatase 2	X	X	**55**	**SOX4**	SRY (sex determining region Y)-box 4	X	
**6**	**DBI**	diazepam binding inhibitor			**56**	**ATXN1**	ataxin 1	X	
**7**	**ENO3**	enolase 3		X	**57**	**FBLN1**	fibulin 1	X	
**8**	**PPP1R1A**	protein phosphatase 1, regulatory (inhibitor) subunit 1A			**58**	**PSAP**	prosaposin		
**9**	**FABP3**	fatty acid binding protein 3		X	**59**	NDRG2	NDRG family member 2		
**10**	COX6A2	cytochrome c oxidase subunit VIa polypeptide 2	X		**60**	**COL1A2**	collagen, type I, alpha 2		
**11**	**MN1**	meningioma			**61**	**SPARC**	secreted protein, acidic, cysteine-rich (osteonectin)		
**12**	**FHL3**	four and a half LIM domains 3		X	**62**	**CHRNB1**	cholinergic receptor, nicotinic, beta 1 (muscle)		X
**13**	FEZ2	fasciculation and elongation protein zeta 2			**63**	**ZFP36L1**	zinc finger protein 36, C3H type-like 1	X	
**14**	**TFRC**	transferrin receptor (p90, CD71)		X	**64**	**CCNG2**	cyclin G2	X	
**15**	**LMOD1**	leiomodin 1		X	**65**	**MTHFD2**	methylenetetrahydrofolate dehydrogenase		
**16**	SEPW1	selenoprotein W			**66**	**ZFP36L2**	zinc finger protein 36, C3H type-like 2	X	
**17**	**TSPAN8**	tetraspanin 8			**67**	**CALM1**	calmodulin 1 (phosphorylase kinase, delta)	X	
**18**	**HRASLS**	HRAS-like suppressor	X		**68**	**CYP26B1**	cytochrome P450, family 26, subfamily B, polypeptide 1		
**19**	COX8A	cytochrome c oxidase subunit VIIIA	X		**69**	**CCT2**	chaperonin containing TCP1, subunit 2 (beta)		
**20**	GMPR	guanosine monophosphate reductase			**70**	**RYR3**	ryanodine receptor 3		
**21**	LMCD1	LIM and cysteine-rich domains 1			**71**	**COL3A1**	collagen, type III, alpha 1	X	
**22**	NDUFA3	NADH dehydrogenase (ubiquinone) 1 alpha subcomplex, 3, 9 kDa	X		**72**	NRAP	nebulin-related anchoring protein		X
**23**	COX6B1	cytochrome c oxidase sub VIb polypeptide 1	X		**73**	**PDGFC**	platelet derived growth factor C		
**24**	MYH2	myosin, heavy chain 2, skeletal muscle, adult		X	**74**	**ETS2**	v-ets erythroblastosis virus E26 oncogene homolog 2		
**25**	ATP5J	ATP synthase, H+ transporting, mitochondrial Fo complex, subunit F6			**75**	**VIM**	vimentin		
**26**	NDUFB3	NADH dehydrogenase 1 beta subcomplex, 3	X		**76**	**PRNP**	prion protein		
**27**	RPL3L	ribosomal protein L3-like			**77**	**DMPK**	dystrophia myotonica-protein kinase		X
**28**	C14orf2	chromosome 14 open reading frame 2	X		**78**	**ADAMTS1**	ADAM metallopeptidase with thrombospondin type 1 motif, 1		
**29**	NDUFB4	NADH dehydrogenase 1 beta subcomplex, 4,	X		**79**	**SLC7A6**	solute carrier family 7 (cationic amino acid transporter, y+ system), member 6	X	
**30**	ART3	ADP-ribosyltransferase 3		X	**80**	**RHOB**	ras homolog gene family, member B	X	
**31**	MYOM2	myomesin (M-protein) 2, 165 kDa		X	**81**	**ARPP21**	cAMP-regulated phosphoprotein, 21 kDa		
**32**	COX6C	cytochrome c oxidase subunit VIc	X		**82**	**COL1A1**	collagen, type I, alpha 1		
**33**	MKNK2	MAP kinase interacting serine/threonine kinase 2	X		**83**	**EZR**	ezrin		X
**34**	COX7C	cytochrome c oxidase subunit VIIc	X		**84**	**HDAC4**	histone deacetylase 4		X
**35**	FKBP3	FK506 binding protein 3, 25 kDa			**85**	**CYR61**	cysteine-rich, angiogenic inducer, 61		
**36**	G0S2	G0/G1switch 2			**86**	**C10orf116**	chromosome 10 open reading frame 116	X	
**37**	COX7A1	cytochrome c oxidase subunit VIIa polypeptide 1	X		**87**	**ANXA1**	annexin A1		X
**38**	COX5B	cytochrome c oxidase subunit Vb	X		**88**	**MYL5**	myosin, light chain 5, regulatory		X
**39**	MEFV	Mediterranean fever		X	**89**	**EEF1A1**	eukaryotic translation elongation factor 1 alpha 1		X
**40**	NDUFAB1	NADH dehydrogenase 1, alpha/beta subcomplex	X		**90**	**RRAD**	Ras-related associated with diabetes		
**41**	MRPL33	mitochondrial ribosomal protein L33	X		**91**	**EEF1A1**	eukaryotic translation elongation factor 1 alpha 1		
**42**	NDUFA6	NADH dehydrogenase 1 alpha subcomplex 6	X		**92**	**MYH3**	myosin, heavy chain 3, skeletal muscle, embryonic		X
**43**	NDUFA4	NADH dehydrogenase1 alpha subcomplex, 4	X		**93**	**CDKN1A**	cyclin-dependent kinase inhibitor 1A (p21, Cip1)		
**44**	ATP5G1	ATP synthase, H+ transporting, mitochondrial Fo complex, subunit C1 (subunit 9)			**94**	**ACTC1**	actin, alpha, cardiac muscle 1		X
**45**	HOXA10	homeobox A10	X		**95**	**MYH8**	myosin, heavy chain 8, skeletal muscle, perinatal		X
**46**	MYOT	myotilin		X	**96**	**MYH8**	myosin, heavy chain 8, skeletal muscle, perinatal		X
**47**	PPP1R3C	protein phosphatase 1, regulatory (inhibitor) subunit 3C			**97**	**CDC42EP3**	CDC42 effector protein (Rho GTPase binding) 3		X
**48**	ATPIF1	ATPase inhibitory factor 1			**98**	**NNMT**	nicotinamide N-methyltransferase		
**49**	MGST3	microsomal glutathione S-transferase 3	X		**99**	**GADD45A**	growth arrest and DNA-damage-inducible, alpha		
**50**	VEGFA	vascular endothelial growth factor A			**100**	**CHRNA1**	cholinergic receptor, nicotinic, alpha 1 (muscle)		X

a MT: mitochondrial genes;

b SM: skeletal muscle-specific genes.

### Network Analysis of the Discriminant Genes

In order to go in depth into the mutual relations between the selected genes, the correlation structure connecting the 100 best discriminant genes was represented as a network, in which the nodes are genes connected by edges.

**Figure 4:**
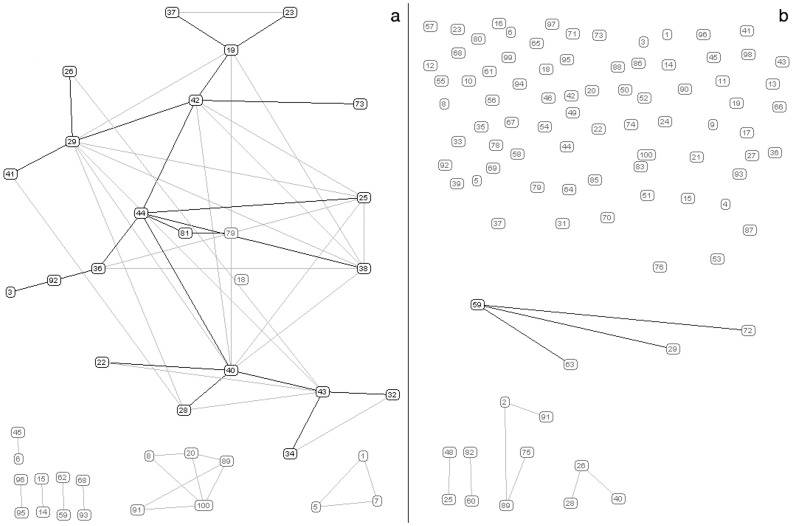
Gene regulation networks. Graphical representation of the inter-gene connection existing in the ALS group (a) and controls (b). The correspondence between the numerical labels and the gene ontology annotation is displayed in table 3.

The graphical representation of the gene networks observed in the two experimental groups (figure 4), clearly showed that genes that were isolated in controls, formed a highly inter-connected sub-network in the ALS group. The correspondence between the numerical labels and the gene ontology annotation is displayed in table 3. For ALS patients, the AD of the network resulted to be 1.16. The network obtained from the normal group had many isolate nodes (83) and an AD as low as 0.2. The widest disease-related sub-network (formed by 22 genes) was mainly formed by mitochondrial genes, while the small sub-networks corresponded to ACTN3- and CHRNA1-correlated genes (figure 4, table 3).

### Biological Interpretation of the Networks

The DAVID-based functional analysis of genes connected in the ALS network, allowed identifying the over-represented functional categories in the list. Table 4 shows the categorization of the genes according to the GO terms. The top biological processes represented in the network (generation of precursor metabolites and energy; striated muscle contraction) were clearly connected to the muscle metabolic activity due to the contractile function (table 4). The molecular function categories indicated that genes interconnected in the ALS network shared a role in oxidative metabolism and in muscle structure definition. Finally, according to the cellular component ontology annotation of the network, a large number of networking genes in the ALS group encoded protein located in the intracellular compartment, either in the mitochondrion or in the myosin complex (table 4).

**Table 4:** DAVID functional analysis.

BIOLOGICAL PROCESS		
Term	Count	FDR
GO:0006936∼muscle contraction	11	<0,001
GO:0006941∼striated muscle contraction	7	<0,001
GO:0003012∼muscle system process	12	<0,001
GO:0006091∼generation of precursor metabolites and energy	18	<0,001
GO:0006119∼oxidative phosphorylation	8	0.0035
GO:0006120∼mitochondrial electron transport, NADH to ubiquinone	6	0.0086
GO:0042773∼ATP synthesis coupled electron transport	6	0.0361
GO:0042775∼mitochondrial ATP synthesis coupled electron transport	6	0.0361
GO:0015980∼energy derivation by oxidation of organic compounds	8	0.0444
**CELLULAR COMPONENT**	**Count**	**FDR**
**Term**		
GO:0044429∼mitochondrial part	16	0.0019
GO:0005739∼mitochondrion	21	0.0035
GO:0030016∼myofibril	8	0.0047
GO:0005747∼mitochondrial respiratory chain complex I	6	0.0054
GO:0045271∼respiratory chain complex I	6	0.0054
GO:0030964∼NADH dehydrogenase complex	6	0.0054
GO:0005859∼muscle myosin complex	5	0.0061
GO:0016460∼myosin II complex	5	0.0092
GO:0031090∼organelle membrane	20	0.0158
GO:0030017∼sarcomere	7	0.0285
GO:0016459∼myosin complex	6	0.0474
**MOLECULAR FUNCTION**		
**Term**		
**Category**	**Count**	**FDR**
GO:0015078∼hydrogen ion transmembrane transporter activity	9	<0,001
GO:0008307∼structural constituent of muscle	7	<0,001
GO:0015077∼monovalent inorganic cation transmembrane transporter activity	9	<0,001
GO:0015002∼heme-copper terminal oxidase activity	7	<0,001
GO:0016675∼oxidoreductase activity, acting on heme group of donors	7	<0,001
GO:0004129∼cytochrome-c oxidase activity	7	<0,001
GO:0016676∼oxidoreductase activity, acting on heme group of donors, oxygen as acceptor	7	<0,001
GO:0008092∼cytoskeletal protein binding	15	0.0058
GO:0022890∼inorganic cation transmembrane transporter activity	9	0.0082
GO:0050136∼NADH dehydrogenase (quinone) activity	6	0.0111
GO:0008137∼NADH dehydrogenase (ubiquinone) activity	6	0.0111
GO:0003954∼NADH dehydrogenase activity	6	0.0111
GO:0016655∼oxidoreductase activity, acting on NADH or NADPH, quinone or similar compound as acceptor	6	0.0212
GO:0016651∼oxidoreductase activity, acting on NADH or NADPH	7	0.0217

Consistently, Kegg pathway mapping indicated that the most represented pathway was the “Oxidative phosphorylation”, with a FDR = 3.5×10^−07^, and fifteen included genes (namely NDUFA4, NDUFB3, NDUFB4, NDUFA3, COX7A1, NDUFA6, COX8A, NDUFAB1, COX7C, ATP5G1, COX5B, COX6C, COX6B1, COX6A2, ATP5J). These data further indicated that mitochondrial activity is significantly affected in the ALS muscle.

## Discussion

This study attempted to provide a systemic view of the human ALS muscle expression profile, in terms of networks based on mutual correlations between intervening genes. A homogeneous patient group was recruited in this study, which comprised ALS patients with a comparable clinical background and disease stage. This homogeneity was reflected by the clear-cut segregation of patients and controls into two groups, based on MDS and PCA. This segregation also suggested that the gene expression profiles observed in the ALS group reflected the extent of skeletal muscle damage, rather than the site of muscle biopsy (either deltoid or quadriceps muscle). Pradat and colleagues previously analyzed the gene expression changes occurring in skeletal muscle from ALS patients in different stages of the disease [30]. Actually, 11 out of the 38 most significant transcripts described in that study were also present in our gene list. In particular we noted the upregulation of the myogenic factor 4 (MYOG) and the concomitant increase in the expression of the acetylcholine receptor subunits (CHRNA1), known to be under the transcriptional control of MYOG [31]. These genes were coherently upregulated in ALS muscles of both human patients and mice, reflecting the response of muscle to the increasing loss of innervation [30]. It is noteworthy that acetylcholine receptor expression has already been used for monitoring the effects of therapy on disease progression in the ALS mice [32].

Also, the small GTP-binding protein RRAD, involved in the modulation of cytoskeleton remodeling and inhibition of voltage-gated calcium channel activity, and GADD45A, a cell cycle inhibitor involved in the apoptosis of atrophying muscle fibers, were upregulated in both the datasets presented in this study and in that by Pradat and colleagues. Moreover, ACTN3, HDAC4, PFKP, whose expression correlated with deltoid muscle injury in ALS patients [30], resulted significantly modulated in our data. In particular, ACTN3 is both the top down-regulated gene and the top-discriminating gene (i.e. the gene that best discriminates between ALS group and controls in the network analysis). On the whole, this independent reproduction of data on an additional sample set, indicates the robustness of the experimental design, along with a confirmation of the key features of the human ALS muscle gene expression profile.

In addition, this study provided original insights into the coordinated regulation of gene expression and its perturbation occurring in the human skeletal muscle of ALS patients, as a result of the systems biology approach employed in the analysis of gene regulatory networks. This allowed hypothesizing previously unknown links between genes showing similar expression trends in ALS muscles compared to controls. In particular, the increased inter-gene connections, observed in the ALS group, should reflect the predicted universal effect of stress condition on biological systems, described by Gorban and colleagues [33]. The gene regulatory network, correlating genes that share common regulation within a biological system, tends to be constrained by the stressful event into a much more correlated model. Looking at the biological functions of the strongly connected genes of the ALS patients, a large number of mitochondrial genes belonging to the oxidative phosphorylation pathway were represented, along with two smaller networks including ACTN3 and CHRNA1. Distinct evidence has recently indicated that mitochondrial content, shape and function correlate with muscle wasting [9], [34], [35]. The gene networks observed in this study could confirm a sort of recognition of a ‘crisis area’ correspondent to mitochondrial activity, Z-line organization and CHRNA1 related cluster.Mitochondrial function, shape and number are closely related to muscle size and integrity. In particular, after denervation of the tibialis anterior muscle, genes typical of fast fibers were downregulated, whereas those typical of slow fibers were upregulated. These changes in gene expression appear to be coordinated in the direction of a fast-to-slow transformation [36]. Consistently, the expression profiles of denervated muscles revealed the molecular signature of a reduced metabolic activity [37].

On this regard, Romanello and colleagues demonstrated that impaired mitochondrial function might activate signals that trigger muscle atrophy in mice, inducing a condition of energy unbalance through the AMP -activated protein kinase (AMPK) signaling [9].

Moreover, the connection between PRKAR1A and FOXO1, observed in this study, may provide some hints towards the delineation of the molecular events associated to human muscle atrophy. PRKAR1A encodes a regulatory subunit of the cAMP-dependent protein kinase, which has been previously demonstrated to accumulate in the NMJ [38]. FOXO1 belongs to the forkhead family of transcription factors, playing a key role in the regulation of skeletal muscle mass. In particular, muscle-specific overexpression of FoxO1 is sufficient to cause skeletal muscle atrophy *in vivo* [39].

Also, TRIM32, a ubiquitin ligase, was highly correlated with both PRKAR1A and FOXO1 in this study. Recently Cohen and colleagues demonstrated that Trim32 ubiquitynates thin filaments (actin, tropomyosin, troponins) and Z-band components (α-actinin), promoting their degradation, during fasting-induced atrophy in mice [40]. It is worth noticing that TRIM32 is not differentially expressed between ALS patients and controls, although being significantly connected with PRKAR1A and FOXO1 in this study. It has been previously shown that, despite its role, Trim32 expression does not increase in fasting-induced atrophy [41].

The gene regulatory network observed in the ALS group, included also a small subnetwork of downregulated genes: ACTN3, ENO3 and FBP2. ACTN3 is one of the four human alpha-actinin isoforms, whose expression was directly associated to the disease progression in human ALS muscles [30]. ENO3 is a muscle-specific enolase, catalyzing the conversion of 2- phosphoglycerate into 2-phosphoenolpyruvate, whose deficiency has been associated to a metabolic myopathy [42]. FBP2 is a muscle-specific regulatory enzyme of glyconeogenesis; biphosphatases (FBPase) are located on both sides of the Z line, in the isotropic regions of myocytes, where they bind strongly to α-actinin, as demonstrated through co-sedimentation experiments [43].

This evidence supports the hypothesis that glyconeogenic enzymes in striated muscle form a metabolic complex on both sides of Z line.

On the whole, the results obtained in this study, supported by some of the most recent literature data, could pave the way to future targeted studies focusing on the functional link between genes involved in metabolic pathways and muscle contractility.

## Supporting Information

Figure S1 **qPCR validation.** Real-time PCR validation. Results of real-time PCR on selected genes. Relative quantity values (RQ) obtained in qPCR are compared to fold changes obtained with microarray analysis (µA). Results for all genes are statistically significant (p<0.01).
(TIF)

Table S1 (TXT)
